# Label-Free White Blood Cell Classification Using Refractive Index Tomography and Deep Learning

**DOI:** 10.34133/2021/9893804

**Published:** 2021-07-30

**Authors:** DongHun Ryu, Jinho Kim, Daejin Lim, Hyun-Seok Min, In Young Yoo, Duck Cho, YongKeun Park

**Affiliations:** ^1^Department of Physics, Korea Advanced Institute of Science and Technology (KAIST), Daejeon 34141, Republic of Korea; ^2^KAIST Institute for Health Science and Technology, KAIST, Daejeon 34141, Republic of Korea; ^3^Department of Health Sciences and Technology, Samsung Advanced Institute For Health Sciences and Technology, Sungkyunkwan University, Seoul 06355, Republic of Korea; ^4^Department of Health and Safety Convergence Science, Korea University, Seoul 02841, Republic of Korea; ^5^Department of Laboratory Medicine and Genetics, Samsung Medical Center, Sungkyunkwan University School of Medicine, Seoul 06351, Republic of Korea; ^6^Tomocube, Inc., Daejeon 34051Republic of Korea; ^7^Department of Laboratory Medicine, Seoul St. Mary’s Hospital, College of Medicine, The Catholic University of Korea, Seoul 06591, Republic of Korea; ^8^Stem Cell & Regenerative Medicine Institute, Samsung Medical Center, Seoul 06531, Republic of Korea

## Abstract

*Objective and Impact Statement*. We propose a rapid and accurate blood cell identification method exploiting deep learning and label-free refractive index (RI) tomography. Our computational approach that fully utilizes tomographic information of bone marrow (BM) white blood cell (WBC) enables us to not only classify the blood cells with deep learning but also quantitatively study their morphological and biochemical properties for hematology research. *Introduction*. Conventional methods for examining blood cells, such as blood smear analysis by medical professionals and fluorescence-activated cell sorting, require significant time, costs, and domain knowledge that could affect test results. While label-free imaging techniques that use a specimen’s intrinsic contrast (e.g., multiphoton and Raman microscopy) have been used to characterize blood cells, their imaging procedures and instrumentations are relatively time-consuming and complex. *Methods*. The RI tomograms of the BM WBCs are acquired via Mach-Zehnder interferometer-based tomographic microscope and classified by a 3D convolutional neural network. We test our deep learning classifier for the four types of bone marrow WBC collected from healthy donors (n=10): monocyte, myelocyte, B lymphocyte, and T lymphocyte. The quantitative parameters of WBC are directly obtained from the tomograms. *Results*. Our results show >99% accuracy for the binary classification of myeloids and lymphoids and >96% accuracy for the four-type classification of B and T lymphocytes, monocyte, and myelocytes. The feature learning capability of our approach is visualized via an unsupervised dimension reduction technique. *Conclusion*. We envision that the proposed cell classification framework can be easily integrated into existing blood cell investigation workflows, providing cost-effective and rapid diagnosis for hematologic malignancy.

## 1. Introduction

Accurate blood cell identification and characterization play an integral role in the screening and diagnosis of various diseases, including sepsis [1-3], immune system disorders [4, 5], and blood cancer [6]. While patient’s blood is examined with regard to the morphological, immunophenotypic, and cytogenetic aspects for diagnosing such diseases [7], the simplest yet the most effective inspection in the early stages of diagnosis is a microscopic examination of stained blood smears obtained from peripheral blood or bone marrow aspirates. In a standard workflow, medical professionals create a blood-smeared slide, fix, and stain the slide with chemical agents such as hematoxylin-eosin and Wright-Giemsa stains, followed by the careful observation of blood cell alternations and cell count as per specific diseases. This not only requires time, labor, and associated costs but also is vulnerable to the variability of staining quality that depends on the staining of trained personnel.

To address this issue, several label-free techniques for identifying blood cells have recently been explored, including multiphoton excitation microscopy [8, 9], Raman microscopy [10-12], and hyperspectral imaging [13, 14]. Each method exploits the endogenous contrast (e.g., tryptophan, Raman spectra, and chromophores) of a specimen with the objective of visualizing and characterizing it without using exogenous agents; however, these modalities require rather complex optical instruments with demanding system alignments and long data acquisition time. More recently, quantitative phase imaging (QPI) technologies that enable relatively simple and rapid visualization of biological samples [15-18] have been utilized for various hematologic applications [19-21]. By measuring the optical path length delay induced by a specimen and by reconstructing a refractive index using the analytic relation between the scattered light and sample, QPI can identify and characterize the morphological and biochemical properties of various blood cells.

Recent advances in artificial intelligence (AI) have suggested unexplored domains of QPI beyond simply characterizing biological samples [22]. As datasets obtained from QPI do not rely on the variability of staining quality, various machine learning and deep learning approaches can exploit uniform-quality and high-dimensional datasets to perform label-free image segmentation [23, 24], classification [25-32], and inference [33-39]. Such synergetic approaches for label-free blood cell identification have also been demonstrated, which are of interest to this work [25, 26, 28, 40-43]. However, these often necessitate manual extraction of features for machine learning or do not fully utilize the high-complexity data of three-dimensional (3D) QPI, possibly improving the performance of deep learning.

**Figure 1:**
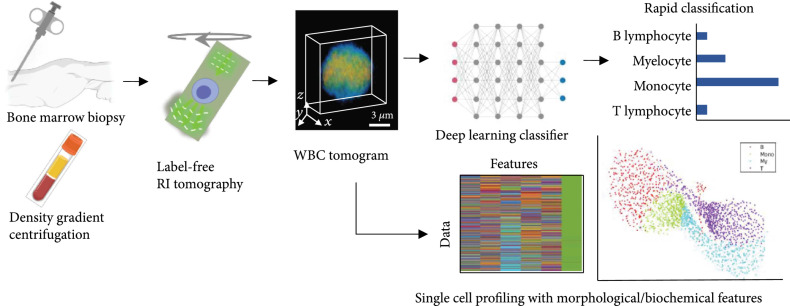
Overview of label-free bone marrow white blood cell (WBC) assessment. Bone marrow WBCs obtained from a minimal process of density gradient centrifugation are tomographically imaged without any labeling agents. Subsequently, individual refractive index (RI) tomograms can be accurately (>95%) and rapidly (<1 s) classified via an optimized deep-learning classifier along with the analysis of morphological/biochemical features such as cellular volume, dry mass, and protein density.

In this study, we leverage optical diffraction tomography (ODT), a 3D QPI technique, and a deep neural network to develop a label-free white blood cell profiling framework (Figure 1). We utilized ODT to measure the 3D refractive index of a cell, which is an intrinsic physical property, and extract various morphological/biochemical parameters of the RI tomograms such as cellular volume, dry mass, and protein density. Subsequently, we use the optimized deep learning algorithm for accurately classifying WBCs obtained from the bone marrow. To test our method, we performed two classification tasks for a binary differential (myeloid and lymphoid, >99% accuracy) and a four-group differential (monocyte, myelocyte, B lymphocyte, and T lymphocyte, >96% accuracy). We demonstrate the representation learning capability of our algorithm using unsupervised dimension reduction visualization. We also compare conventional machine learning approaches and two-dimensional (2D) deep learning with this work, verifying the superior performance of our 3D deep learning approach. We envision that this label-free framework can be extended to a variety of subtype classifications and has the potential to advance hematologic malignancy diagnosis.

## 2. Results

### 2.1. Morphological Biochemical Properties of WBCs

**Figure 2:**
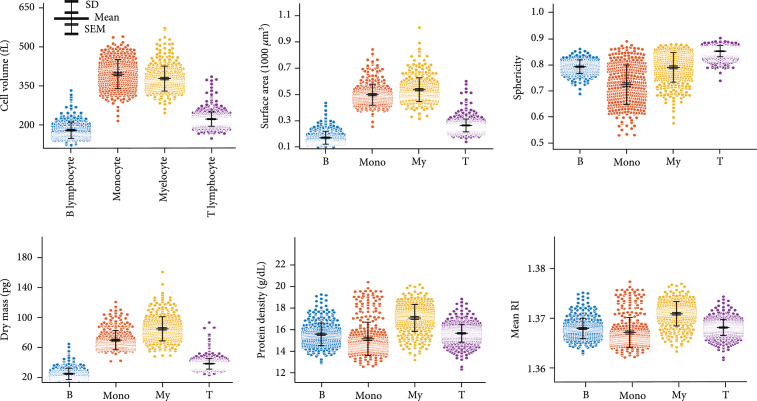
Quantitative analysis of morphological (cell volume, surface area, and sphericity), biochemical (dry mass and protein density), and physical (mean RI) parameters. The protein density is directly related to the mean RI. The scatter plots represent the entire population of the measured tomograms. SD: standard deviation; SEM: standard error of the mean; B: B lymphocyte; Mono: monocyte; My: myelocyte; T: T lymphocyte.

We first quantified morphological (cell volume, surface area, and sphericity), biochemical (dry mass and protein density), and physical (mean RI) parameters of bone marrow WBCs and statistically compared them in Figure 2. These parameters can be directly obtained from the 3D RI tomograms (see Materials and Methods). The mean and standard deviation of cell volumes for B lymphocytes, monocytes, myelocytes, and T lymphocytes are 185.05±34.86 fL, 422.79±61.31 fL, 404.19±53.10 fL, and 232.31±30.35 fL, respectively; in the same order, the surface areas are 169.39±49.21 *μ*m^2^, 498.29±80.60 *μ*m^2^, 536.59±91.01 *μ*m^2^, and 263.35±48.84 *μ*m^2^; the sphericities are 0.794±0.026, 0.725±0.076, 0.792±0.057, and 0.854±0.022; the dry masses are 26.44±8.13 pg, 75.38±14.01 pg, 91.80±17.51 pg, and 41.19±7.88; the protein densities are 15.58±1.05 g/dL, 15.15±1.55 g/dL, 17.09±1.26 g/dL, and 15.67±0.83 g/dL; the mean RIs are 1.368±0.002, 1.367±0.003, 1.371±0.002, and 1.368±0.001.

Several observations are noteworthy. First, the mean cellular volume, surface, and dry mass of lymphoid cells (B and T lymphocytes) are smaller than those of myeloid cells (monocytes and myelocytes). The morphological properties of the B and T cells are directly related to the dry mass because we assumed that the cells mainly comprised proteins (0.2 mL/g). Furthermore, we observed that the sphericity of the lymphoid group was larger than that of the myeloid group. The B and T lymphocytes, which commonly originate from small lymphocytes, have one nucleus and spherical shapes; the monocyte and myelocyte cells have a more irregular morphological distribution. Overall, the standard deviations of all parameters except the mean RI for the myeloid group were larger than those of the lymphoid group, indicating the larger morphological and biochemical variability in the myeloid group. Finally, it is challenging to accurately classify the four types of WBCs by simply comparing these parameters (e.g., thresholding). Although lymphoid cells could be rather differentiated from myeloid cells based on the cellular volume, surface area, or dry mass at the “population” level, the overlapped population on all parameters across the two groups still impedes the accurate classification at the “single-cell” level. More importantly, classifying within lymphoid or myeloid (e.g., classification of B and T) would be even more difficult as their statistical parameters are very similar.

### 2.2. Three-Dimensional Deep Learning Approach for Accurate Classification

**Figure 3:**
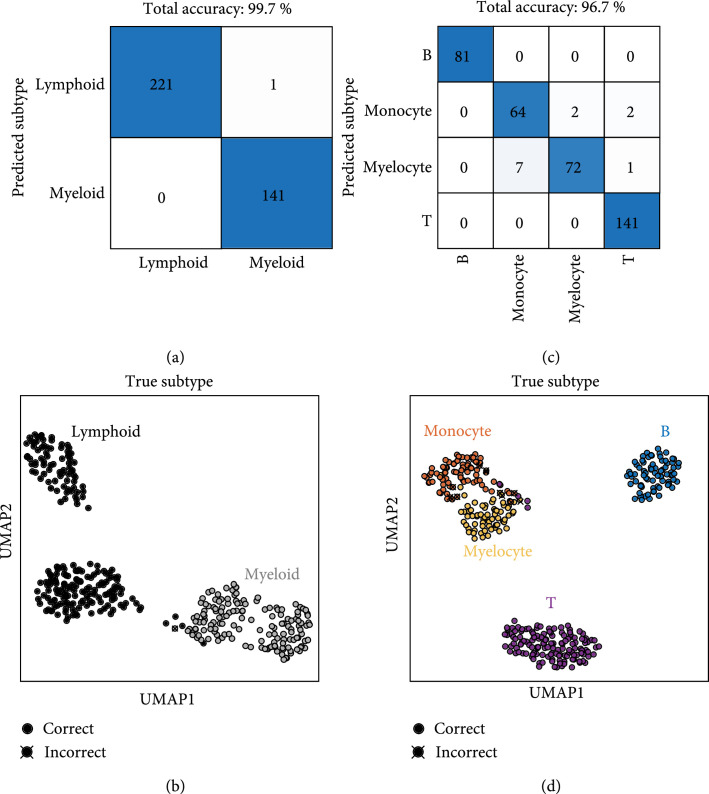
Classification results using our approach. Two classifiers are independently designed for (a, b) the binary classification of lymphoid and myeloid and (c, d) the classification of B/T lymphocytes, monocyte, and myelocyte, respectively. The powerful learning capability of the optimized classifiers is illustrated via uniform manifold approximation and projection (UMAP).

To achieve an accurate classification of the WBCs at the single-cell level, we designed and optimized a deep-learning-based classifier by fully exploiting the ultrahigh dimension of WBC RI voxels, presenting two independent classification results in Figure 3. We first tested the 3D deep-learning classifier for the binary classification of lymphoid and myeloid cell groups. For the unseen test dataset, the binary classification accuracy of the trained algorithm was 99.7%, as depicted in Figure 3(a). Remarkably, only one lymphoid-group cell was misclassified as a myeloid cell. The powerful learning capability of our network is visualized by the unsupervised dimension reduction technique, UMAP (see Materials and Methods, Section 4.6) in Figure 3(b). High-dimensional features were extracted from the last layer of the second downsampling block of the trained network. A majority of test data points are clearly clustered, while few data points of myeloids and lymphoids are closely located. This indicates that our well-trained algorithm not only extracts various features that differentiate the two groups but also finely generates a complex decision boundary for such unclustered data points for accurate classification. It is also interesting that roughly four clusters were generated through deep learning, although we trained the algorithm to classify the two groups, implying that the learning capacity of our algorithm would be sufficient for the classification of more diverse subtypes (see also Figure S1).

We also tested another deep neural network that classifies the four types of WBCs with a test accuracy of 96.7%. The predictions of the trained algorithm for the four different subtypes are shown in Figure 3(c). Our algorithm correctly classified all B lymphocytes, while monocyte and myelocyte groups were misclassified, and a few T lymphocytes were misclassified into myeloid group cells. UMAP visualization was also performed for this result (Figure 3(d)). The B cell cluster is clearly distant from the remaining clusters. Meanwhile, the monocyte and myelocyte clusters are closely located, thereby providing the most cases of misclassification. A few data points of T cells were also found near these clusters. Despite the significantly similar statistics across the four subtypes as confirmed in the previous section, our algorithm is capable of extracting useful visual features from millions of voxels and generating a sophisticated decision boundary, achieving high test accuracy for the unseen data. It is also noteworthy that a small subset (e.g., 10% of the training set) could be sufficient to achieve a test accuracy of 90% or more (Figure S2).

### 2.3. Conventional Machine Learning and 2D Deep Learning

**Figure 4:**
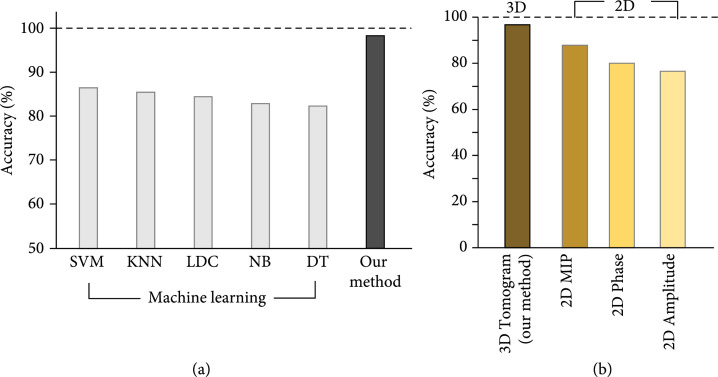
Conventional approaches in comparison with the present method. (a) Comparison of classification accuracy between conventional machine learning algorithms that require hand-crafted features and our 3D deep learning. (b) Performance of deep learning with various input data. SVM: support vector machine; KNN: k-nearest neighbors algorithm; LDC: linear discriminant classifier; NB: naïve Bayes; DT: decision tree; MIP: maximum intensity projection.

For further validation of our method, we benchmarked our 3D deep learning approach against conventional machine learning (ML) approaches that require handcrafted features. First, widely used ML algorithms, such as support vector machines, k-nearest neighbors, linear discriminant classifiers, naïve trees, and decision trees, were performed and compared with our method for the four-type classification (see Materials and Methods, Section 4.5). The test accuracies for the five algorithms along with our method are shown in Figure 4(a). While the machine learning algorithms do not exceed 90% accuracy, our method achieved more than 96% test accuracy, as confirmed in the previous section. We reasoned that the six parameters obtained from 3D RI tomograms would not be sufficient for the conventional algorithms to generate an accurate classification boundary of the 3D deep network, although additional feature engineering or extractions may help improve the performance of the machine learning algorithms. Yet, the machine learning approaches are capable of classifying the lymphoid and myeloid groups of which morphological and biochemical parameters are differentiated enough (Figure S3).

A 2D deep neural network that processes various 2D input data was also explored. We used a 2D maximum intensity projection (MIP) image that can be directly obtained from a 3D RI tomogram, 2D phase, and amplitude to train the deep network. The classification accuracies for the four different inputs are shown in Figure 4(b). While the 2D MIP obtained directly from the 3D tomogram achieved an accuracy of 87.9%, which is the closest to our approach, the network trained with 2D phase and amplitude presented accuracies of 80.1% and 76.6%. These results suggest that it is important to fully utilize 3D cellular information with an optimal 3D deep-learning classifier for accurate classification.

## 3. Discussion

In this study, we have demonstrated how synergistically utilizing optical diffraction tomography and deep learning can be employed to profile bone marrow white blood cells. With minimal sample processing (e.g., centrifugation), our computational framework can accurately classify white blood cells (lymphoids and myeloids with >99% accuracy; monocytes, myelocytes, B lymphocytes, and T lymphocytes with >96% accuracy) and their useful properties such as cellular volume, dry mass, and protein density without using any labeling agents. Moreover, the presented approach, capable of extracting optimal features from captured RI voxels, outperformed the well-known machine learning algorithms requiring handcrafted features and deep learning with 2D images. The powerful feature learning capability of our deep neural networks was demonstrated via UMAP, an unsupervised dimension reduction technique. We anticipate that this label-free workflow that does not require laborious sample handlings and domain knowledge can be integrated into existing blood tests, enabling the cost-effective and faster diagnosis of related hematologic disorders.

Despite successful demonstrations, several future studies need to be conducted for our approach to be applicable in clinical settings. First, diverse generalization tests across imaging devices and medical institutes should be performed. In this study, we acquired the training and test datasets from only one imaging system installed at a single site. Exploring diverse samples and cohort studies at multiple sites using several imagers would strengthen the reliability of our approach. Second, the current data acquisition rate needs to be improved. While we manually obtained and captured the tomograms of WBCs within the limited field of view of high numerical aperture (30×30 *μ*m^2^, 1.2 NA), the use of a motorized sample stage well synchronized with a high-speed camera can significantly boost the data acquisition rate. The microfluidic system might be integrated into the current system to improve the imaging speed; however, tomographically imaging rapidly moving samples as accurately as static ones would be challenging. Ultimately, we need to extend the classification to other types of bone marrow blood cells for diagnosing various hematologic diseases such as leukemia. As conducted in manual cell differentials by medical professionals, standard cell types to be counted, such as promyelocytes, neutrophils, eosinophils, plasma cells, and erythroid precursors, should be included the training dataset for clinical uses. As the diversity of cell types and the complexity of information increases, it may require the significant tuning of our network hyperparameters or even redesigning of the network architecture.

## 4. Materials and Methods

### 4.1. Sample Preparation and Data Acquisition

**Figure 5:**
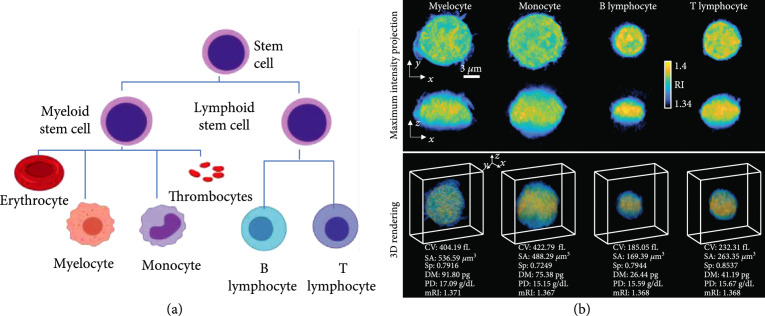
WBC lineage relevant to our study and representative RI tomograms. (a) Two myeloid cells and two lymphoid cells are used to demonstrate our framework. (b) Representative RI tomograms are visualized through two different perspectives of maximum intensity projection and 3D isosurface rendering. CV: cellular volume; SA: surface area; Sp: sphericity; DM: dry mass; PD: protein density; mRI: mean RI.

Four types of white blood cells were collected from the bone marrow of ten healthy donors investigated: myelocyte, monocyte, B lymphocyte, and T lymphocyte (Figure 5(a)). We also note that we obtained 1-2 types of WBCs per donor owing to a limited amount of blood sample and low magnetic-activated cell sorting yield (refer to Figure S4). In WBC lineage, myelocytes and monocytes stem from myeloids; B and T lymphocytes stem from lymphoids.

First, the bone marrow was extracted via a needle biopsy. To isolate mononuclear cells (MC), the bone marrow was diluted in phosphate-buffered saline (PBS; Welgene, Gyeongsan-si, Gyeongsangbuk-do, Korea) in a 1 : 1 ratio, centrifuged using Ficoll-Hypaque (d =1.077, Lymphoprep™; Axis-Shield, Oslo, Norway), and washed twice with PBS. Next, magnetic-activated cell sorting (MACS) was performed to obtain the four types of WBCs from the isolated MC. CD3+, CD19+, and CD14+ MACS microbeads (Miltenyi Biotec, Germany) were used to positively select T lymphocytes, B lymphocytes, and monocytes. Myelocyte cells were isolated through the negative selection of CD14 and positive selection of CD33. For optimal sample density and viability, we prepared each isolated sample in a mixed solution of 80% Roswell Park Memorial Institute (RPMI) 1640 medium, 10% heat-inactivated fetal bovine serum (FBS), and 10% 100 U/mL penicillin and 100 *μ*g/mL streptomycin (Lonza, Walkersville, MD, USA). While imaging, we kept the samples in an enclosed iced box.

A total of 2547 WBC tomograms were obtained using our imaging system. After sorting out noisy dataset (e.g., moving cells, coherent noises) [44], the number of datasets for myelocytes, monocytes, B lymphocytes, and T lymphocytes was 403, 379, 399, and 689, respectively. For deep learning, we randomly shuffled the entire dataset and split the training, validation, and test set by a 7 : 1 : 2 ratio.

The representative 3D RI tomograms for each subtype are visualized as a maximum intensity projection image and a 3D-rendered image in Figure 5(b). The morphological and biochemical parameters can be directly computed from the measured RI tomograms, which is further explained in the next section.

### 4.2. Subjects and Study Approval

The study was approved by the Institutional Review Board (No. SMC 2018-12-101-002) at the Samsung Medical Center (SMC). All blood samples were acquired at the SMC between November 2019 and June 2020 (see Figure S4). We selected patients suspected of having lymphoma who underwent bone marrow biopsy and were all diagnosed with normal bone marrow at the end.

### 4.3. Quantification of Morphological/Biochemical Properties

Six parameters calculated from a reconstructed tomogram were obtained: cellular volume, surface area, sphericity, dry mass, protein density, and mean refractive index. First, the cellular volume and surface area were directly acquired by thresholding the tomogram. The voxels with RI values higher than the thresholding RI value were segmented; the thresholding value was 1.35, considering a known medium RI of approximately 1.33 and experimental noises. Sphericity was calculated by relating the obtained surface area S and volume V as follows: $\text{sphericity}=\pi^{1/3}\cdot {\left(6\;V\right)}^{2/3}/S$.

Next, the biochemical properties such as protein density and dry mass were obtained from RI values via a linear relation between the RI of a biological sample and its local concentration of nonaqueous molecules (i.e., proteins, lipids, and nucleic acids inside cells). Considering that the proteins are major components and have mostly uniform values, protein density can be directly converted from RI values as follows: $n=n_{0}+\alpha C$, where n and $n_{0}$ are the RI values of a voxel and the medium, respectively, and α is the refractive index increment (RII). In this study, we used an RII value of 0.2 mL/g. The total dry mass can be calculated by integrating the protein density over the cellular volume.

### 4.4. Deep Learning Classifier

**Figure 6:**
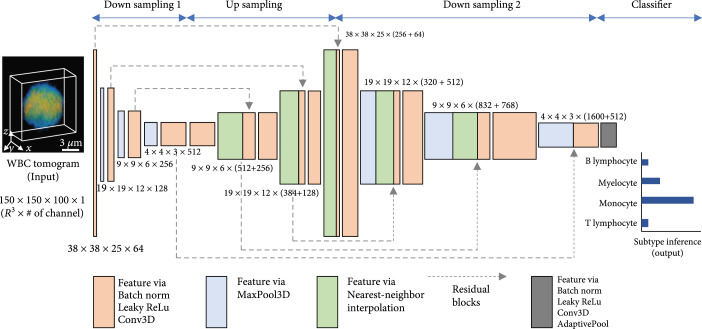
Three-dimensional deep neural network for WBC classification. RI tomograms are processed to extract distinguishing features of each WBC through a convolutional neural network comprising downsampling and upsampling operations, leading to the subtype inference at the end.

We implemented a deep neural network to identify the 3D RI tomogram of myelocytes, monocytes, T lymphocytes, and B lymphocytes. Our convolutional neural network, inspired by FISH-Net [45], comprises two downsampling (DS) blocks, an upsampling (US) block, and a classifier at the end (Figure 6). The first DS block, comprising batch norm, leaky ReLu, 3D convolution, and 3D max pooling, extracts various features at low resolution. Next, the US block upsamples using nearest-neighbor interpolation and refines the features obtained from the previous block via residual blocks that connect all three DS and US blocks together. These residual blocks help the improved flow of information across different layers and relax the well-known vanishing gradient. The second DS block processes not only the previous features from the US block but also the features transmitted via the residual blocks with batch norm, leaky ReLu, 3D convolution, and 3D max pooling. Ultimately, the extracted features, after being processed by the classifier comprising batch norm, leaky ReLu, 3D convolution, and adaptive 3D pooling, are set to the most probable subtype for the classification task.

Our network was implemented in PyTorch 1.0 using a GPU server computer (Intel® Xeon® silver 4114 CPU and 8 NVIDIA Tesla P40). We trained our network using an ADAM optimizer [46] (learning rate=0.004, momentum=0.9, and learning rate decay=0.9999) with a cross-entropy loss. The learnable parameters were initialized by He initialization. We augmented the data using random translating, cropping, elastic transformation, and adding Gaussian noise. We trained our algorithm with a batch of 8 for approximately 18 hours. We selected our best model at 159 epochs and monitored the validation accuracy based on MSE. The prediction time for a single cell (150×150×100 voxels) takes approximately 150 milliseconds using the referred computing specifications. Though the inference time depends on a batch and voxel size, our approach is expected to take hundreds of milliseconds in most of practical cases.

### 4.5. Conventional Machine Learning Classifier

To compare our deep learning approach with existing machine learning approaches, we implemented support vector machine (SVM), k-nearest neighbors (KNN), linear discriminant classifier (LDC), naïve Bayes (NB), and decision tree (DT) algorithms to classify the four types of WBCs using six extracted features (cellular volume, surface area, sphericity, dry mass, protein density, and mean RI). Six binary-SVM models with error-correcting output codes were trained to make a decision boundary for the four classes. For the KNN classifier, k=5 was chosen. All machine learning algorithms were implemented using MATLAB.

### 4.6. Uniform Manifold Approximation and Projection (UMAP) for Visualization

To effectively visualize the learning capability of our deep neural network, we employed a cutting-edge unsupervised dimension reduction technique known as uniform manifold approximation and projection (UMAP) [47]. UMAP constructs the high-dimensional topological representations of the entire dataset and optimizes them into low-dimensional space (e.g., two dimensions) to be as topologically similar as possible. We extracted the learned features from the last layer of the second downsampling block immediately before entering the classifier block and applied UMAP to demonstrate the feature learning capability of the trained classifier in two dimensions.

## Supplementary Materials

Supplementary Materials Figure S1: UMAP visualization of the binary classification. Figure S2: stability of our classifier depending on the size of training set. Figure S3: conventional approaches for the binary classification of lymphoids and myeloids. Figure S4: donor information. Figure S5: tomographic system and reconstruction.
