# Supplementary material for: Label-Free White Blood Cell Classification Using Refractive Index Tomography and Deep Learning
Source: BME Front. 2021 Jul 30;2021:9893804. doi: 10.34133/2021/9893804 (PMC10521749; doi:10.34133/2021/9893804)
Supplement: Supplementary Materials — Figure S1: UMAP visualization of the binary classification. Figure S2: stability of our classifier depending on the size of training set. Figure S3: conventional approaches for the binary classification of lymphoids and myeloids. Figure S4: donor information. Figure S5: tomographic system and reconstruction. [file 9893804.f1.docx]

**Supplementary Materials**

**Figure S1. UMAP visualization of the binary classification.** (Left) The binary classification of lymphoid and myeloid (Figure 3 from the main text). (Right) The same UMAP labeled by the four subtypes (T lymphocyte, B lymphocyte, monocyte, and myelocyte). We postulate that our deep neural network, trained for the binary classification, has sufficient learning capacity for the four-subtypes classification.

**Figure S2. Stability of our classifier depending on the size of training set.** We re-trained a four-type classifier using randomly sampled data (50%, 25%, 10%) from the whole training set and tested on the identical test set (n=370). It is worth noting that the lymphoid lineage (B, T lymphocyte) is robustly classified even with the 10% of the training set. Yet, the classification accuracy for the myeloid lineage (monocyte, myelocyte) diminished. The four classifiers were trained using the same hyperparameters as described in the main text.

**Figure S3. Conventional approaches for the binary classification of lymphoids and myeloids.**

**Figure S4. Donor information.** Donated WBC subtypes and donation dates per each donor are described. This table provides unsplit data that includes training, validation, and test set.

**Optical diffraction tomography**

Our 3D imaging system is a commercialized optical diffraction tomography microscope (also known as holotomography) using Mach–Zehnder interferometry and a digital micromirror device (DMD) for high-speed angle-scanned illumination (HT-2H, Tomocube, Inc., South Korea) [41]. The schematics of its optical setup are depicted in Figure S1(a). A diode-pumped solid-state laser beam (532-nm wavelength, MSL-S-532-10mW, CNI laser, China) was used for the sample beam and reference beam, split by a fiber coupler (OZ optics, Canada). The sample beam, angle-scanned by a DMD (DLP65300FYE, Texas Instruments, USA), impinges on a sample after passing through a condenser objective lens (UPLASAPO 60XW, Olympus Inc., Japan). The scattered signal, 4-f-relayed by an objective lens (UPLASAPO 60XW, Olympus Inc., Japan) and a tube lens, interferes with the reference beam transmitted via a beam splitter. After being filtered by a linear polarizer, 49 interferograms were captured by a complementary metal-oxide-semiconductor camera (FL3- U3-13Y3M-C, FLIR Systems, Inc., USA), as shown in Figure S1(b).

Next, the amplitude and phase images are retrieved from the measured interferograms using a phase-retrieval algorithm that utilizes spatial filtering [42] and the Goldstein phase unwrapping method [43]. Based on the Fourier diffraction theorem with Rytov approximation [44], a sample’s 3D RI distribution is reconstructed from the retrieved amplitude and phase images. To fill in the side-scattering signal not collected owing to the limited numerical apertures of objective lenses, a regularization algorithm utilizing a non-negative constraint is employed [45]. The theoretical resolutions of the ODT system are 110 nm (lateral) and 330 nm (axial), according to the Lauer criterion [46]. The data acquisition time for the 49 interferograms is approximately 500 ms, and it requires typically several seconds to reconstruct a regularized tomogram using a standard personal computer. The reconstruction time for the tomogram can be further reduced by a high-end graphics processing unit. All these customized image reconstruction codes were implemented in MATLAB (MathWorks, USA).

**Figure S5. Tomographic system and reconstruction.** (a) Optical setup of ODT. (b) Fourty-nine interferograms with angle-scanned illuminations, each complex field (amplitude and phase) retrieved from each interferogram, and the 3D RI distribution reconstructed from the fourty-nine complex field images. L: Lens. FC: Fiber coupler. DMD: Digital micromirror device. Obj: Objective lens. M: Mirror. BS: Beam splitter. P: Linear polarizer. CAM: Complementary metal-oxide-semiconductor camera.
